# Co-design of a Smartphone App for People Living With Dementia by Applying Agile, Iterative Co-design Principles: Development and Usability Study

**DOI:** 10.2196/24483

**Published:** 2022-01-14

**Authors:** Sarah Fox, Laura J E Brown, Steven Antrobus, David Brough, Richard J Drake, Francine Jury, Iracema Leroi, Adrian R Parry-Jones, Matthew Machin

**Affiliations:** 1 Global Brain Health Institute Trinity College Dublin Dublin Ireland; 2 Manchester Centre for Health Psychology Division of Psychology and Mental Health, School of Health Sciences, Faculty of Biology, Medicine and Health University of Manchester Manchester United Kingdom; 3 Division of Informatics, Imaging & Data Sciences University of Manchester Manchester United Kingdom; 4 Division of Neuroscience and Experimental Psychology Faculty of Biology, Medicine and Health University of Manchester Manchester United Kingdom; 5 University of Manchester Manchester United Kingdom; 6 Manchester Centre for Clinical Neurosciences Salford Royal NHS Foundation Trust Salford United Kingdom; 7 Division of Cardiovascular Sciences University of Manchester Manchester United Kingdom

**Keywords:** agile, dementia, co-design, cognition, mHealth, patient public involvement, software development, mobile phone

## Abstract

**Background:**

The benefits of involving those with lived experience in the design and development of health technology are well recognized, and the reporting of co-design best practices has increased over the past decade. However, it is important to recognize that the methods and protocols behind patient and public involvement and co-design vary depending on the patient population accessed. This is especially important when considering individuals living with cognitive impairments, such as dementia, who are likely to have needs and experiences unique to their cognitive capabilities. We worked alongside individuals living with dementia and their care partners to co-design a mobile health app. This app aimed to address a gap in our knowledge of how cognition fluctuates over short, microlongitudinal timescales. The app requires users to interact with built-in memory tests multiple times per day, meaning that co-designing a platform that is easy to use, accessible, and appealing is particularly important. Here, we discuss our use of Agile methodology to enable those living with dementia and their care partners to be actively involved in the co-design of a mobile health app.

**Objective:**

The aim of this study is to explore the benefits of co-design in the development of smartphone apps. Here, we share our co-design methodology and reflections on how this benefited the completed product.

**Methods:**

Our app was developed using Agile methodology, which allowed for patient and care partner input to be incorporated iteratively throughout the design and development process. Our co-design approach comprised 3 core elements, aligned with the values of patient co-design and adapted to meaningfully involve those living with cognitive impairments: end-user representation at research and software development meetings via a patient proxy; equal decision-making power for all stakeholders based on their expertise; and continuous user consultation, user-testing, and feedback.

**Results:**

This co-design approach resulted in multiple patient and care partner–led software alterations, which, without consultation, would not have been anticipated by the research team. This included 13 software design alterations, renaming of the product, and removal of a cognitive test deemed to be too challenging for the target demographic.

**Conclusions:**

We found patient and care partner input to be critical throughout the development process for early identification of design and usability issues and for identifying solutions not previously considered by our research team. As issues addressed in early co-design workshops did not reoccur subsequently, we believe this process made our product more user-friendly and acceptable, and we will formally test this assumption through future pilot-testing.

## Introduction

### Background

In January 2019, the National Health Service published its long-term plan, setting out key ambitions for the next 10 years. One of the most ambitious targets of this plan was in the field of digital technology, with a vision toward increasing care at home using remote monitoring and digital tools [1]. This move will likely be expedited by the need for social distancing brought about by the COVID-19 pandemic. Therefore, with the impetus and growing necessity for distance health care, it is important to consider how this new type of service will meet the needs of patients. A way to ensure that new technologies are usable, acceptable, and tailored toward the patients they aim to support is to ensure that the patients themselves are central to the design and development process. Co-design offers a way to ensure that new technologies and interventions are tailored to patient needs [2]. Indeed, there is growing support for the benefits of co-design in health care [3] but less evidence as to how these approaches can be tailored to the needs of diverse patient populations [4].

Within the context of software development, co-design can be defined as a process that draws on the shared creativity of software developers and people not trained in software working together [5]. To this end, special attention is given to involving end users and ensuring that their input as experts through experience is central to the design process and that their specific needs are understood and met [5-7]. This is in line with existing literature suggesting that integrating patient voice with software development is achievable and can provide valuable feedback to improve the intuitive design and usability of software outcomes [8,9].

In dementia research, patient and public involvement (PPI) and co-design is still a developing field [10], although it has been suggested to confer benefits to research outcomes, researchers, and members of the public who play a part in the process [11,12]. The capacities, needs, and preferences of those living with cognitive impairments can be diverse [13]. Therefore, standard co-design and PPI methodologies often need to be adapted to suit this population. This may be particularly important in the field of digital technology and software development, as studies suggest that there is utility and an appetite for assistive technology for older people and those living with cognitive impairments. However, despite there being motivation for older people to use digital technologies, barriers exist around usability and lack of experience [14-17].

An area in which digital technology can help with the care and management of dementia is through the monitoring of cognitive change and variability, which is an issue that is considered important for this population [18,19]. For instance, many people with dementia experience worsening cognitive and neuropsychiatric symptoms in later periods of the day, a phenomenon known as *sundowning* [20,21]. Current practice bases the diagnosis of dementia on a combination of clinical history, biomarker detection, and examination, of which cognitive testing is a key part [22]. However, conventional cognitive assessments cannot detect short-term fluctuations in cognition that might be relevant to understanding or managing individuals’ cognitive, functional, and behavioral symptoms.

Recent developments in computerized cognitive testing have made it possible to measure microlongitudinal patterns of cognitive function [23]. However, although these tools have been tested in cognitively healthy older adults [14], they have not yet been used in populations with cognitive impairment. Furthermore, these tasks were designed for use on large, touchscreen tablet devices and have not yet been adapted for use on smaller, more mobile devices, such as smartphones, which are used by an increasing number of older people [23]. Therefore, there is an impetus to adapt such tasks for use on smartphone devices and meet the needs of those living with clinical conditions that affect their cognitive abilities [24].

Despite the diverse and divergent lived experiences of those living with dementia, software apps are rarely designed with this patient population in mind [15]. It is even rarer to find software codeveloped alongside those living with dementia [4]. This can result in poorer quality technology that can be difficult to use for those living with dementia [25]. However, research indicates that those living with dementia have an interest in assistive technology and are capable of using touchscreen technology [17,26]. Therefore, we approached the adaptation of microlongitudinal computerized cognitive tests to the needs of people living with cognitive impairments through an iterative Agile process with patient co-design at its center.

Agile software development focuses on collaboration with users and rapid software deployment [27]. Scaling tests to a mobile device requires regular input and development iterations from end users, with an understanding that direct translation between devices may be unsuitable. The Agile methodology is best suited to such projects where requirements may not be clearly defined at the outset and emerge over time [28]. This is especially relevant in this case, where iterative co-design workshops spaced throughout the development process meant that the final product was not clearly defined early in the process and, instead, emerged based on consultations with experts through experience via regular workshops.

### Objectives

In this paper, we describe how we modified co-design approaches to involve members of the public living with dementia and their care partners in the production of a smartphone app. Although we worked specifically with people with dementia, the principles could be applied to other patient groups who do not find it easy to engage with standard co-design approaches. We also explain the benefits of the Scrum development methodology as a way of integrating user feedback into the design and development process.

## Methods

### PPI and Co-design: Theoretical Framework

#### Overview

Public involvement in research is defined by the National Institute for Health Research’s INVOLVE as research being conducted *with* or *by* members of the public rather than *to*, *about*, or *for* them. Tambuyzer et al [29] also recognize that, given the heterogeneity of research protocols and patient populations, involvement is not a *one-size-fits-all* concept and is better defined by values rather than protocols. These values include participation in decision-making and giving contributors some control and responsibility over research outcomes, active involvement that goes beyond consultation or receiving information, involvement in a range of activities, being recognized as experts by experience, and collaboration with professionals.

Working alongside individuals living with cognitive impairments necessitates a tailored approach to involvement and co-design. Therefore, it is necessary to balance facilitating meaningful involvement alongside being mindful of individuals’ capacity, capability, and preferences.

Therefore, we approached the challenge of co-design alongside individuals with cognitive impairments by adopting the following three methodological steps: (1) end-user representation at research and software development meetings via a patient proxy; (2) equal decision-making power for all stakeholders based on their expertise; and (3) continual user consultation, user-testing, and feedback.

#### Step 1: End-user Representation

On the basis of the combination of a short timescale for app development, limitations in the availability of clinical advisors, and a desire to reduce unnecessary burden on contributors living with dementia and their care partners, we chose to represent the patient or public voice at research group meetings via a proxy. Our proxy was a PPI officer who worked alongside our research group. They were responsible for developing and facilitating co-design workshops and representing end users at research group meetings. This ensured that the patient voice was represented in all important decisions and was given equal weight as the voice of other research team members.

#### Step 2: Equality of Expertise

Input from those with lived experience of cognitive impairments was integral to the development of this app. Therefore, those involved were encouraged to input into all the elements of the design process. To this end, input from those with lived experience led to 13 design alterations across the life of the project (listed in the following sections). Feedback from co-design workshops also led to the removal of 1 cognitive test, which was deemed too challenging for those living with dementia, and rebranding of the app.

#### Step 3: Continued Input

**Figure 1:**
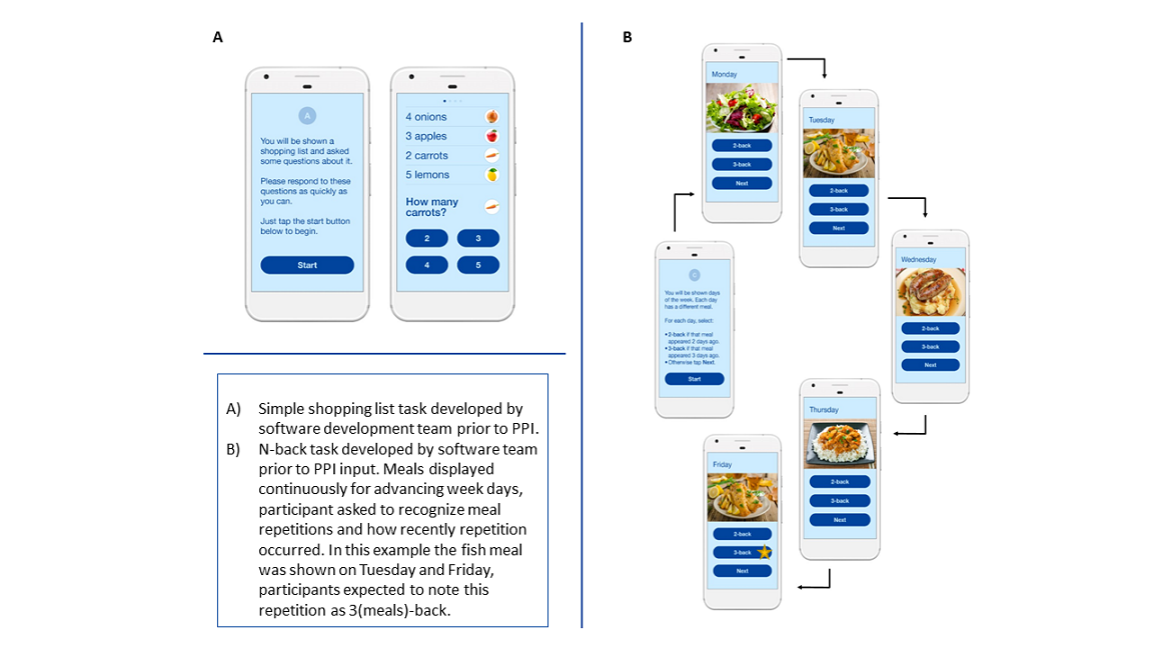
(A) Simple test of cognitive processing speed and (B) a more cognitively demanding tests of working memory developed by the software development team before patient and public involvement input. PPI: patient and public involvement.

**Figure 2:**
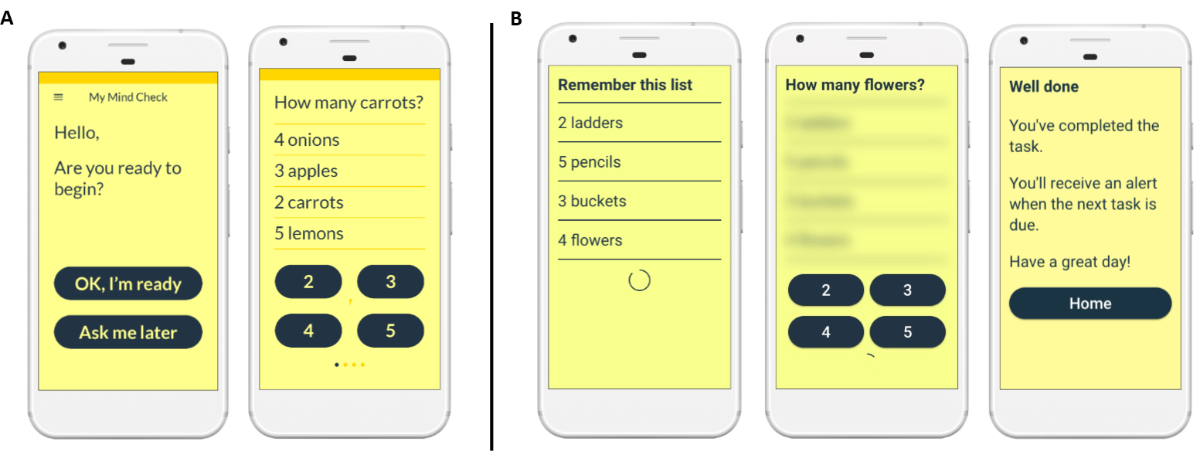
(A) Redesigned shopping list task and (B) new shopping list+ task following first patient and public involvement workshop.

**Figure 3:**
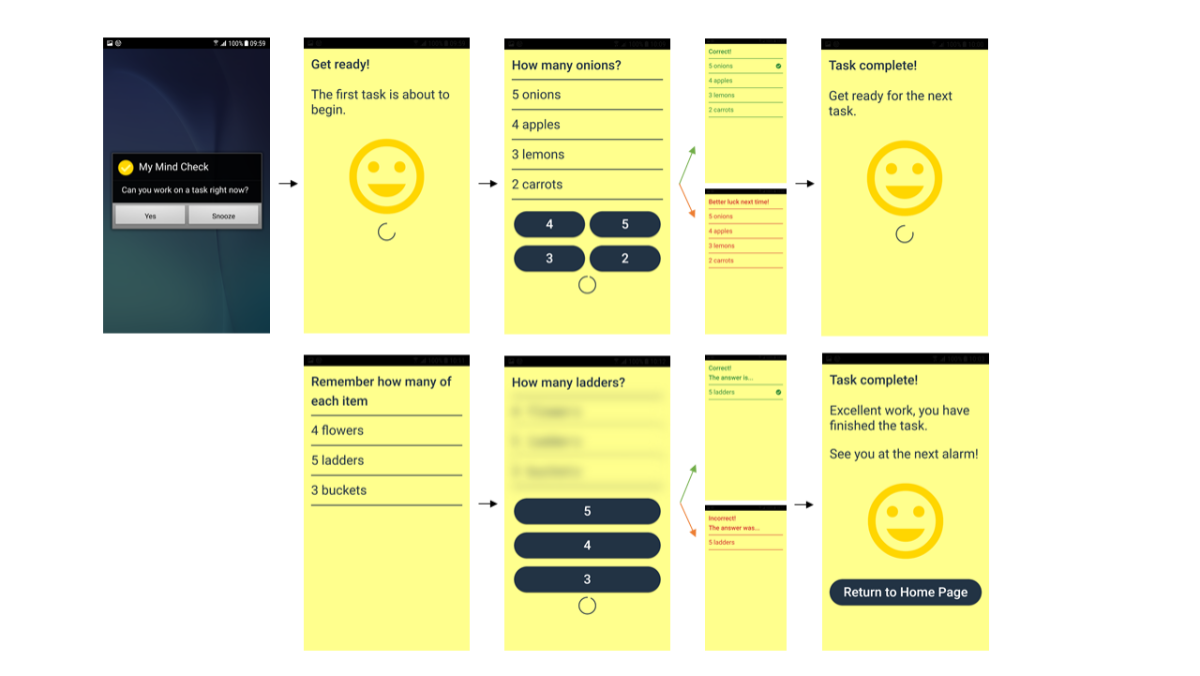
Final software alterations following second patient and public involvement workshop showing flow through the app.

Following the development of an initial prototype app, which was designed to act as a scaffolding example app for use in the first co-design workshop, all subsequent software development sprints were based on end-user feedback. This ensured that any emerging design or software features were reviewed and modified by end users before being added to the following sprint. The extent of the end-user modifications adopted in the creation of this app can be visualized by comparing Figure 1 (the research team’s prototype app) with Figure 2 (alterations made following our first co-design workshop) and Figure 3 (the final product based on feedback from 4 co-design workshops).

### PPI, Co-design Process, and Methodology

#### Overview

A total of 4 co-design workshops were run collaboratively with community dementia support groups and were tailored to those living with cognitive impairments. Workshops were planned around familiar venues and, in some cases, to coincide with existing support group meetings. The materials used were dementia-friendly [30] and in line with INVOLVE recommendations [31]; the budget was ring-fenced to cover attendee travel, attendance fees, and refreshments.

Participants of workshops 1 and 2 comprised a mix of individuals living with a dementia diagnosis and current and past care providers of people living with dementia (workshop 1: 5/7, 71% with dementia and 2/7, 29% current or past carers; workshop 2: 3/6, 50% with dementia and 3/6, 50% current or past carers). Participants were recruited from 2 local dementia support groups following informal visits and presentations from the members of the research team. Approximately 30% (3/10) of the participants (1/3, 33% living with dementia, and 2/3, 67% current or past carers) attended both workshops 1 and 2.

Workshops 1 and 2 adopted a similar format: each workshop lasted approximately 2 hours and included (1) lunch and informal ice-breaker conversations; (2) a short, accessible project discussion and feedback; (3) introduction and testing of a visual working prototype; and (4) the collection of informal one-to-one and group feedback on the prototype. Workshop 1 also included an activity in which participants were encouraged to discuss their views on and responses to candidate words and phrases for the app’s name. This was achieved via a discussion of flashcards containing keywords associated with the cognitive testing app (eg, cognition, test, training, research, brain, *e*, memory, noggin, and mobile) alongside our prototype name *Health-e-Mind*. This discussion generated the name *MyMindCheck*, which was considered meaningful and acceptable to workshop attendees. This name was later presented to the participants of workshop 2, 7 months after workshop 1, and was received positively.

These workshops were designed to involve patients in the co-design of the *MyMindCheck* app rather than being structured research or focus groups. Therefore, feedback from participants was not treated as research data. Consistent with common involvement practice [32], participant feedback was collected as written field notes by 2 workshop facilitators (direct quotes were not included); these notes were collated, and key points were identified and fed back to the research team.

Workshops 3 and 4 took place 2 months after workshop 2 and spanned a week-long period of user-testing. Participants were recruited by the research team from a local community dementia support group with a focus on technology; these were older individuals with current or past experience of supporting someone living with dementia (n=4 current or past carers). This group was targeted as we expected individuals attending a technology-focused group to be inclined to take part in our week-long user-testing phase.

Potential participants from this group were approached during one of the group’s regular meetings, and the project was introduced, and the app was demonstrated. From this meeting, 4 individuals consented to the 7-day testing period, whereas 4 declined, citing time commitments as a barrier to participation. We returned to the group the following week to distribute phones preloaded with the *MyMindCheck* app, a short instruction manual, and optional paper diaries. The paper diaries were used as an aide-memoir for participants to record their day-to-day experiences using the app.

Workshop 4 took place after the week-long user-testing period and comprised a short informal discussion regarding participants’ experiences of using the software. Participants were asked to comment not only on their own experience with the software but also on its suitability for someone living with a dementia diagnosis. Paper diaries were referred to during this discussion as a memory prompt; data from these diaries were not stored or analyzed further outside this workshop.

Feedback from each workshop was reviewed and discussed by the project team shortly after each workshop. This resulted in an agreed set of changes for the subsequent shippable products. As with any feedback of this nature, the project team prioritized changes based on both the effort required to implement and the likely impact on the end user.

#### The Software Development Process

The *MyMindCheck* app was developed using a co-design approach [5-7] involving three key groups of stakeholders: the research team (including clinical input), the target user group (people living with dementia and individuals with direct experience of caring for those with dementia), and the software development team. The software team adopted the Scrum framework for development [33]. Scrum is a modern, Agile software development methodology that fits well with the co-design approach used to develop this app. It focuses on the regular delivery of working software (*shippable products*) to users and depends on user feedback throughout the software development.

Scrum uses sprints, which are timeboxed development efforts, usually 1 to 4 weeks in duration [33]. The software team used 3-week sprints for this project, as this presented a suitable balance between the need to be able to respond flexibly to changing requirements and the delivery of sufficient functionality within each sprint. Sprint planning sessions were attended by members of the research and software development team, including a PPI specialist (research team member) who facilitated public workshops and acted as a customer proxy during these planning sessions. The customer proxy acted as the Scrum product owner in this instance and was responsible for being the voice of the customer. In Scrum, the product owner is responsible for defining and prioritizing the requirements for the product, which, in this case, was the *MyMindCheck* app [34]. The PPI specialist was chosen as the product owner as they worked closely with the PPI participants to capture the requirements for the app.

Visual working prototypes were used during the initial PPI workshop events. These prototypes allowed users to see interactive screens that portrayed key design elements and the flow through the app and were produced by a user experience designer embedded in the software team. This enabled early testing with the research team and target user group without requiring significant investment in software development.

The initial prototypes were based on a validated computerized cognitive task, in which participants were presented with a short list of grocery items (eg, carrots) and a quantity for each [23]. Participants were then asked to report the quantity of a given item (Figure 1). This task had previously been validated as a measure of cognitive processing speed in community-living older people [23]. An additional task was also included to place a higher demand on memory, which was based on clinical input. This task was based on an N-back test of working memory [35] and presented participants with a meal for each day of the week. When a participant was shown a meal that had previously been seen, they needed to recall how many days *back* the meal was first seen (Figure 1).

Before the first PPI workshop, research team members, who had experience in working with people with cognitive impairment, reviewed the prototypes. They suggested modifications to simplify these tasks, making them more visually appealing and quicker to navigate to maintain user engagement. These modifications included the following:

1. The addition of images to both tasks
2. Start screens containing instructions on how to complete the tasks
3. Simplification of the second, harder, N-back task by introducing images of meals and the use of the days of the week, as these were familiar items (Figure 1)
4. The team also generated the provisional name
  Health-e-Mind
  for the prototype app

These prototypes were then presented for review to the target user group during co-design workshops arranged by the team’s public involvement lead. On the basis of initial feedback on the visual working prototypes, the software team began the development of version 1 of the app. This process continued in an iterative manner for each of the key components of the app.

#### Learning From the Approach

Throughout the project, the research and software development teams met on an approximately monthly basis to review the approach being taken. This enabled improvements to be made to the process within the project and resulted in some key learning points that could be applied to future projects.

## Results

### Initial Prototype and Feedback From Workshop 1

The initial prototype comprised a shopping list task and an N-back task (Figure 1).

Much of the participant feedback collected during workshop 1 correlated poorly with the research team’s prior assumptions. Some of the key feedback from workshop 1 and the actions taken to address this feedback are presented in Table 1.

**Table 1:** Feedback and resulting software modifications from workshop 1.

Item and feedback			Software modification
**Appearance**			
	Testers found pictures to be distracting. Specifically, it was noted that some pictures were confusing (ie, onion and apple looked similar) and that the images shifted focus away from reading written information, making it harder to follow instructions.	From this feedback, the software development team chose to remove images from both tasks.	
	Participants reported that the color scheme (dark blue text on a light blue background) might be inappropriate for those with reading or perceptual difficulties. Black writing on a yellow background was suggested to be optimal for improving reading speed and for assisting people with reading difficulties.	The display was altered to black text on a yellow background.	
**Instructions**			
	Testers noted that detailed introductory text explaining the task was not necessary for the simple shopping list task. Indeed, several participants stated that they skipped reading the introductory message and were still able to perform the task.	The development team removed the introduction text from this task, replacing it with a simple “Are you ready to start <yes>, <no>” structure.	
	It was noted that the screen flow used in the shopping list task left some participants confused. Specifically, several participants felt that displaying the shopping list followed by a probe question was less logical (harder to follow) than displaying the probe question first followed by the shopping list.	Text flow was altered in line with workshop preferences in the next design iteration (Figure 2).	
	The shopping list task relied on measures of task completion time as a proxy for cognition. Therefore, instructions for this task asked participants to complete the task “As quickly as possible.” Workshop participants noted that although they read this instruction, they did not feel a sense of urgency while completing the task, suggesting that they had not remembered it.	To encourage users to complete the shopping list task as quickly as possible, the development team added a circular bar countdown timer to the bottom of the task screen.	
**N-back task**			
	Participants felt that the written explanation for the second, harder (N-back) task was insufficient, and, even after a verbal explanation and demonstration, many were still uncomfortable interacting with this task.	It was decided that the N-back task was too complicated and not fit for purpose. Therefore, the development team removed this task and replaced it with a more memory-intensive variant of the shopping list task, subsequently referred to as shopping list+, in which the shopping list was removed from the screen before and during each probe question (Figure 2).	
**Feedback**			
	Participants were asked whether they would appreciate feedback on their performance on these tasks. Opinions were mixed, with some participants wanting graphed data, or indications of low and high performance, whereas others felt that feedback on poor performance might reduce their motivation to complete future tasks.	It was decided that a generic positive feedback message would be added to the tasks, that is, “Great job, well done.”	
**Name**			
	Participants did not like the name the research team chose for the app—Health-e-Mind. Most were unaware that the e stood for electronic, and 1 individual mentioned that it made him think of drug use. Group feedback on flashcard word association included the following: Brain was seen to be too biological, whereas mind was preferred as this sounded more holistic and accessible. Although some participants were comfortable with the words test and memory, others suggested that these terms may be off-putting and could cause anxiety. It was suggested that the word test could be replaced by check as this sounded less daunting and clinical. Participants also liked the addition of the word my to the name, personalizing the app.	From this feedback, the team chose to change the name to *MyMindCheck*.	

Overall, workshop participants seemed positively disposed to the purpose of the app and said that assuming certain alterations were made, they would be willing to interact with such a program on a subdaily basis.

### Second Prototype and Feedback From Workshop 2

Building on feedback from workshop 1, the software team undertook a second development sprint, updating the original prototype to incorporate feedback from workshop 1, including removal of N-back task and replacement with shopping list+ task (Figure 2).

Feedback from this workshop and actions taken are listed below in Table 2.

**Table 2:** Feedback and resulting software modifications from workshop 2.

Item and feedback		Software modification
**Instructions**		
	For the new shopping list+ task, a number of participants noted that until they reached the screen containing the question and multiple-choice answers, they did not realize that they had to remember both the objects listed and the associated number of items.	This was addressed by altering the prompt used on the first screen of this task to read “Remember how many of each item.”
**Appearance**		
	For the shopping list+ task, several participants were unable to read the entire list of 4 items displayed on the first screen before it timed out and moved on to the probe question.	To address this, the team increased the display duration of the first screen to give users more time to read the instruction and object list. They also reduced the list length from 4 items to 3.
	This version included a countdown timer on both tasks, specifically, a circular bar countdown timer. Although most testers said that they did not notice this timer, they did note that they had been trying to respond quickly. However, 1 tester did say that she noticed the timer and felt stressed about completing the task in time.	The countdown timer remains in the app as a visual cue to complete in a timely manner. However, the timer was altered from a model which showed a finite time counting down to a timer that did not count down to a finite point. It was hoped that this maintained a sense of urgency but would mitigate stress caused by a finite countdown.
**Feedback**		
	This version of the app included a generic positive feedback message after each task that was not linked to performance, that, “Well done.” This was included to avoid user discouragement because of low scores. However, participants did not appreciate being given positive feedback when they were aware that they had performed badly.	Feedback was altered to maintain a positive tone while also remaining performance neutral: “Task complete! You have finished the task. See you at the next alarm.”

In line with feedback from the first workshop, no major objections were raised to the usability and acceptability of the *MyMindCheck* app. Indeed, 1 attendee who stated at the beginning of the workshop that she did not use mobile phones was particularly fast to pick up both tasks and noted at the end of the workshop that she had enjoyed testing the app.

### Final Prototype and Feedback From Workshops 3 and 4

Workshops 3 and 4 aimed to test the software alterations implemented as a result of workshop 2 and to trial new functionality, including prompts and alarms. Feedback from these workshops and the actions taken to address this feedback are listed as follows:

Although most participants complied with the assigned in-app tests, the most common reasons for noncompliance were

1. The alarm was not loud or long enough.
2. Fear of breaking the phone if they took it out of the house.
3. Fatigue at being asked to complete tasks 4 times a day.

To address these concerns, the software team implemented the following modifications:

1. Increased the alarm volume and duration
2. Decided to provide phone cases when using a study phone to reduce fear of dropping or damaging phones
3. Decided to implement further PPI regarding prompt number and frequency before further implementation or testing

One tester also noted in her diary that, for the first 2 days of testing, the phone did not register her responses, and therefore was timing out on the tests. Similar issues had surfaced with other testers to a lesser extent in some of the preceding workshops. On the basis of this feedback and previous observations, it was noted that some participants were holding the response buttons rather than tapping them, perhaps reflecting a level of unfamiliarity with mobile technology among this group. Therefore, to address this issue the software was modified to identify both on-press and on-hold events as valid answers.

Testers were confident using both tasks, although they noted that they found the shopping list+ task harder and that it required more concentration. Testers with experience caring for someone with dementia stated that they believed that these tasks could be completed by someone living with dementia, assuming they had support from a care partner.

## Discussion

### Principal Findings

We used a co-design approach to develop the *MyMindCheck* app involving three key groups of stakeholders: the research team (including clinical input), the target user group (people living with dementia or individuals with direct experience of caring for others with dementia), and the software development team. As patient involvement and co-design in dementia research is in its relative infancy across Europe [10], this study will make an important contribution toward a model of best practice for related research and provide an exemplar for others wishing to adopt and modify this approach. Our report conforms to the Guidance for Reporting Involvement of Patients and the Public-2 international reporting guidelines for PPI [36]. Therefore, findings from this study will be comparable and address concerns raised by some researchers regarding the lack of consistent reporting in co-design research [37], especially in regard to those living with dementia [4].

By adopting Scrum in this context, we were able to realize the benefits of an iterative co-design approach, with the software evolving throughout each of the 4 workshops. In addition, our use of prototype designs in the first 2 workshops provided the team with a low-cost opportunity to receive feedback and evaluate the idea before commencing software development.

Participants in the workshops gave positive feedback about the experience, showed strong engagement during the sessions, and provided constructive comments on the app. Notably, points raised in early workshops did not resurface in the week-long test undertaken by a different participant group at a later stage. This could be because users in the week-long test focused on different aspects of the app. However, it could also suggest that our approach was effective in addressing design issues at an early stage of development.

Although the co-design methodology enabled the team to iteratively develop the app, we still had to overcome several challenges. For instance, it was agreed early on that embedding of an end user (in this case, someone living with a dementia diagnosis into the Scrum team), as per co-design best practice [38], would not be feasible because of the burden that regular meetings could place on those living with a dementia diagnosis and their care partners, as well as the power differentials and communication difficulties associated with involving lay members in technical discussions [39]. Instead, we took the pragmatic approach of running workshops throughout the project to garner regular feedback from the user group and provide end-user representation through a proxy (in our study, the proxy was a public engagement officer who developed and facilitated all co-design workshops). Ideally, the project would have benefited from more regular contact with the end-user group. However, given the vulnerable nature of this group, our approach seemed to be an appropriate compromise, given that this was a fast turnaround, intensive development project.

There were some logistical challenges in running the Agile development using a co-design process. Specifically, recruiting participants for workshops required multiple interactions with community groups to garner interest in the project and plan suitable times and venues for workshops. Therefore, it was necessary to set the date for each workshop several weeks in advance. However, software development does not always run according to the plan, as it is not possible to estimate development tasks with a high degree of accuracy. Therefore, there is a risk that a date could be set for a workshop only for the software not to be ready in time. We mitigated this risk by setting a date for each workshop, which allowed a suitable leeway for any unexpected delays. We also worked with an experienced and established Scrum team, meaning that the estimates could usually be provided with a reasonable level of confidence.

### Conclusions

Given the need for health research, particularly the development of health technology, to be approached in a patient-centered manner [37], we developed a methodology that combines Agile software development with integrated patient co-design. This approach facilitated meaningful user involvement in a manner that was easily manageable by our project team, who were working on a short timescale with budget constraints, a challenge experienced by many developers [40].

We also highlighted several instances where input provided by people with lived experience of dementia helped our team to identify and address usability issues early in the development process, speeding up delivery and reducing software development waste. Our experience evidences how co-design can benefit the software development process and be sustainably tailored to the needs of diverse patient populations [4].

The next step for the *MyMindCheck* app is to undertake a large pilot trial and adaptations to apply to other health conditions with fluctuating cognitive states. Patient groups will continue to be involved throughout this future work to ensure that the developed software is fit for its purpose.

## Abbreviations

PPI: patient and public involvement
